# The Risk Factors of the Alcohol Use Disorders—Through Review of Its Comorbidities

**DOI:** 10.3389/fnins.2018.00303

**Published:** 2018-05-11

**Authors:** Ping Yang, Rui Tao, Chengsen He, Shen Liu, Ying Wang, Xiaochu Zhang

**Affiliations:** ^1^Department of Medical Psychology, Chaohu Clinical Medical College, Anhui Medical University, Hefei, China; ^2^Department of Substance-Related Disorders, Anhui Mental Health Center, Hefei, China; ^3^Key Laboratory of Brain Function and Disease, School of Life Sciences, University of Science and Technology of China, Chinese Academy of Sciences, Hefei, China

**Keywords:** alcohol use disorders (AUDs), comorbidity, major depressive disorder (MDD), schizophrenia, personality disorders (PDs)

## Abstract

Alcohol use disorders (AUDs) represent a severe, world-wide problem, and are usually comorbid with psychiatric disorders, comorbidity increases the risks associated with AUDs, and results in more serious consequences for patients. However, currently the underlying mechanisms of comorbid psychiatric disorders in AUDs are not clear. Studies investigating comorbidity could help us understand the neural mechanisms of AUDs. In this review, we explore three comorbidities in AUDs, including schizophrenia, major depressive disorder (MDD), and personality disorders (PDs). They are all co-morbidities of AUDs with rate of 33.7, 28, and 50–70%, respectively. The rate is significantly higher than other diseases. Therefore we review and analyze relevant literature to explore whether these three diseases are the risk factors of AUDs, focusing on studies assessing cognitive function and those using neural imaging. We found that memory deficits, impairment of cognitive control, negative emotion, and impulsivity may increase an individual's vulnerability to AUDs. This comorbidity may indicate the neural basis of AUDs and reveal characteristics associated with different types of comorbidity, leading to further development of new treatment approaches for AUDs.

## Introduction

Alcohol use disorders (AUDs) are chronic and relapsing illnesses that contribute to around 4% of the global burden of disease (Margolese et al., 2004; Rehm et al., 2009). The prevalence of 12-month and lifetime AUD (DSM-5) has been reported to be 13.9% and 29.1%, respectively (Grant et al., 2016). AUDs increase the risk of many adverse consequences, including injury, disease, death (Barbosa et al., 2010; Dawson and Grant, 2011), and substantial financial cost (Joshua, 2017).

The neural mechanisms of AUDs remain uncertain. According to previous studies, there are multiple possible causes of AUDs, including genetic, psychological, an environment factors (Kendler et al., 2011). However, there are complex factors in identification of genetic risk variants, involved in alcohol-related phenotypes. Until recently, gene finding efforts have mainly focused on candidate genes. Certain factors play a role in alcohol-related phenotypes in addition to genetic factors. For example, disadvantageous life circumstances, including early life stress and stressful life events (e.g., death of a loved one, divorce) (Holgate and Bartlett, 2015). Similar to stress, poor life satisfaction has been associated with alcohol use and heavy drinking (Peltzer and Pengpid, 2016). Individuals with psychiatric disorders often exhibit comorbidity with AUDs (Sher, 2006). The close relationship between psychiatric disorders, such as schizophrenia, mood disorders, and personality disorders, and AUDs suggests that psychiatric disorders may increase the risk of exposure to alcohol, exacerbating other early life risk factors for AUDs (Fink et al., 2016). Studies on the comorbidity of AUDs in psychiatric disorders might be useful to help us understand the neural mechanisms of AUDs. Therefore, it is important to enhance our understanding of these comorbidities.

Alternatively, it is plausible that the AUDs would induce some psychiatric disorders. The early study found that AUDs were related to impulsive behavior, negative affect and weaken self-regulation (Drevets et al., 1997) which in turn caused other mental disorders. Additionally, the self-medication hypothesis suggests that AUDs promote individuals to continue using alcohol to reduce negative emotional and mental symptoms, which predicted greater risk factors for comorbiding with other psychiatric disorders (Abrams et al., 2001).

However, this review focuses on revealing the risk factors of AUD, therefore we present current studies of AUDs and their associated comorbidities, including MDD, schizophrenia, and PDs, with a particular focus on the effect of these disorders on the AUD.

## Schizophrenia and AUDs

Over one-third of patients with schizophrenia meet the criteria for an AUD diagnosis, which is more than three times the prevalence in the population at large (Regier et al., 1990; Green and Brown, 2006). The Epidemiologic Catchment Area (ECA) study found that the co-morbidity rate of the two diseases was as high as 33.7% (Regier et al., 1990). These results suggest that schizophrenia may act as a vulnerability factor to AUDs.

Studies show that significant predictors of comorbid AUDs in patients with schizophrenia are being male, severity of negative symptoms, severity of depression (Meszaros et al., 2011), low educational attainment, previous violent offending, and a family history of substance use disorders (Apantaku-Olajide et al., 2014).

However, there is an important risk factor for relapse in abstinent alcoholics psychological stress, as well as the neural mechanisms, by which stress induces relapse are fairly well established. Chronic alcohol using, especially in stress, results in neuroadaptations. Thus alcoholic patients show dysfunction of stress [e.g., sympathetic adrenomedullary axis (SAM) and hypothalamic pituitary adrenocortical axis (HPA)] pathways, characterized by (for example) dysregulation of the cortisol response (Kreek and Koob, 1998), and/or deficits in emotional regulation (Sinha, 2001). These neuroadaptations may lead to patients showing increases in craving for alcohol in response to stress, and thus being particularly at risk of relapse.

The co-morbidity of AUDs in patients with schizophrenia is associated with greater severity of psychopathology (Margolese et al., 2004) and neurocognitive dysfunction (Manning et al., 2009). Several studies suggest that individuals with schizophrenia and comorbid AUDs have exacerbated impairments in memory, including working memory (Bowie et al., 2005; Potvin et al., 2008), episodic memory (Smith et al., 2011), and verbal learning (Manning et al., 2009).

Volume loss within localized regions of the hippocampus (Sinha, 2001) and subcortical structures, such as the thalamus (Byne et al., 2009), striatum and globus pallidus (Brandt and Bonelli, 2008), can be seen as schizophrenia characteristic.Smith and colleagues (Smith et al., 2011) reported that schizophrenia patients with AUD had a similar a more widespread pattern of hippocampal deformity with inward deformation near the head and tail in the right hemisphere and inward deformations in the left head that extended more medially. Within the hippocampus and subcorticalstructures, experts have suggested earlier that addiction and schizophrenia may have overlapping neurobiological substrates, which may place schizophrenia patients at increased risk for developing a substance use disorder (Green et al., 2002).

In addition developmental dysfunction of the hippocampus also leads to a reward circuit dysfunction gets further validation in a rodent model of preadolescent hippocampal lesioning-the neonatal ventral hippocampal lesion rat (NVHL rat), in which normal hippocampal signaling (particularly to the frontal cortex and the nucleus accumbens) is altered during development (Tseng et al., 2009). NVHL rats not only exhibit a schizophrenia-like phenotype, but they also have a dysregulated reward circuit. Importantly, they also demonstrate increased consumption of alcohol (Jeanblanc et al., 2015). Data from research on this animal model suggest that brain reward circuit dysfunctions increase the vulnerability for substance use in patients with schizophrenia, which combined with potentially accentuated reinforcing properties of the substances, may make patients with schizophrenia especially vulnerable to initiating substance use.

Moreover, individuals at an elevated risk of schizophrenia have been suggested to be particularly vulnerable to the effects of alcohol on the frontal lobe and hippocampus (Welch et al., 2011), which are implicated in memory processes. These neural imaging studies suggest that brain regions associated with memory may also support the neural mechanisms of AUDs. Consistently, Luo and colleagues recently reported that an associative memory retrieval-extinction procedure decreases reinstatement of cocaine and heroin seeking in rats, and heroin craving in humans (Luo et al., 2015). The authors conclude that retrieval-extinction manipulations, started 1 day after cocaine self-administration, decreased reinstatement (cocaine priming), spontaneous recovery, and renewal (context-induced reinstatement) of cocaine seeking.

Additionally, cognitive dysfunction, in particular executive dysfunction (Manning et al., 2009), is another major symptom in schizophrenic patients with comorbid AUDs. This suggests that executive dysfunction is a risk factor of AUDs. In a study of non-alcoholic addiction, individuals with cannabis-related impairment in executive function have been found to have trouble learning and applying the skills required for successful recovery, putting them at an increased risk of relapsing to cannabis use (Crean et al., 2011). Similarly, this may hinder an individual's ability to benefit from behavioral therapies, increasing the risk of relapse to alcohol use (Aharonovich et al., 2008). An array of higher cognitive functions is vital for overriding and inhibiting responses that otherwise would be automatic or require little thought, such as continued substance abuse.

In summary, a number of studies (Smith et al., 2011; Johnson et al., 2013) have shown that schizophrenia results in severe abnormalities in the hippocampus and related brain regions that affect memory and executive function. Disorganized memory and the inability to inhibit craving may be risk factors for inducing alcohol-seeking and subsequent alcohol-dependence.

## Depressive disorders and AUDs

MDD as well as alcohol use disorders (alcohol abuse and alcohol dependence) are widespread in the general population and constitute a huge public health burden worldwide (World Health Organization, 2011). In addition, comorbidity of alcohol use disorders and MDD is high (Boschloo et al., 2011). Studies in the general population show that people with depressive disorders have a 2- to 3-fold increased risk of AUDs (Hasin et al., 2007). With respect to 12-month comorbidity among respondents with a diagnosis of alcohol dependence, 29% of respondents had at least one affective disorder and the most common was major depression (28%) (Burns and Teesson, 2002). It is suggested that MDD is likely to be a pathogenic factor in triggering AUDs. Likewise, chronic drinking may promote depression indirectly as well. For example by contributing to stressful life circumstances (e.g., partner-relationship disruptions) that in turn are known to promote depression (Conner et al., 2009) (See Table 1).

**Table 1:** Summary of some studies on the relationship between the MDD and the AUDs.

**Study**	**MDD predicts AUDs**	**AUDs predicts MDD**	**Male or female**	**Cognitive impairment**
Hogarth et al., 2018	√		Both	Sensitivity of negative mood
Uekermann et al., 2003	√	√	Male	Memory impairment
Dichter et al., 2010	√		Male	Cognitive control
Vasile and Vasiliu, 2017	√	√	Both	Visual spatial ability
Fein and Cardenas, 2017	√		Both	Inhibitory executive control networks
Quervain et al., 2017		√	Both	Short-term memory
Thapinta et al., 2017	√	√	Both	Social cognition
Brown and Shepard, 2016		√	Male	Working memory

The self-medication hypothesis (Kalén et al., 1990) suggests that people with depression misuse alcohol to reduce their distressing symptoms, This theory has been corroborated by another research (Bolton et al., 2006). Studies examining clinical characteristics show that people with comorbid alcohol dependence have more depressive symptoms (Rae et al., 2002). One possible explanation is that distressing circumstances, such as having no partner or feelings of loneliness, in combination with a neurotic personality may increase the likelihood of an individual to reduce distress with alcohol.

Structural and functional neuroimaging (Solomon and Corbit, 1974; Walker et al., 2011) has provided evidence in support of some degree of neuropathological convergence in AUDs and mood disorders. In chronic alcoholics, prefrontal regions show abnormally reduced glucose metabolism or blood flow in medial, dorsolateral, and orbitofrontal regions (Beaunieux et al., 2013). Similarly, in mood disorders, dysregulation of glucose metabolism and blood flow particularly affects the prefrontal cortex (PFC), temporal cortex, and amygdale (Videbech, 2000).

A therapeutic study (Dichter et al., 2010) found that cognitive control of MDD decreased significantly. Cognitive biases, in the context of addiction, can be broadly defined as automatically triggered cognitive processes, such as paying attention to and approaching drug cues. One such bias, attentional bias, is the degree to which attention is drawn to motivationally relevant stimuli as opposed to neutral stimuli. This can be quantified through reaction time tasks and measured in a laboratory setting. Attentional bias for drug cues has been studied extensively (Dependence, 2008) and is (modestly) related to craving (Fadardi and Cox, 2009). Furthermore, attentional bias has been related to relapse and escalation of drug problems. As such attentional bias for drug cues is thought to be an important behavioral marker for (problematic) drug use (Stacy and Wiers, 2010).

Attentional bias to social threats, as an initiator of cognitive distortion, is central to social fear (Dudeney et al., 2015). The role of attentional bias to threat has been implicated in the development, maintenance, and remediation of anxiety pathology (Dudeney et al., 2015). The heightened salience of these cues can “grab” attention leading to drug-seeking, a cascade effect that may not even require conscious awareness of the drug cues (Rose et al., 2008).

Imaging studies show when patients were urged to pay attention to their own heartbeats, they exhibited notable responses in the lateral orbitofrontal cortex (OFC), which found that lateral OFC hyperactivity may be responsible for anxiety-laden cognitions (Hahn et al., 2011). Previous a study has shown that the gray matter volume of the PFC reduced in familial bipolar disorder and familial MDD (Drevets et al., 1997). Another study suggests that patients with MDD have reduced OFC gray matter volumes (Lacerda et al., 2004). Further, the defect of the OFC in MDD may result in attention deviation.

These results suggest that the defect of PFC (especially in the lateral OFC) leading to the deficient cognitive control in MDD possibly increases the risk of developing co-morbid AUDs.

Neurobiological studies also support the self-medication hypothesis. In the context of the opponent-process theory (Solomon and Corbit, 1974), activation of the associated receptor is proposed to underpin the “negative” component, such as dysphoria and MDD (Yan and Roth, 2004), which drives drug-seeking behavior and consumption. Consistent with this theory, in preclinical models of AUD, antagonism of the κ-opioid receptor function, with nalmefene for example (Quelch et al., 2017), reduces alcohol consumption (Walker et al., 2011).

Furthermore, a previous study (Uekermann et al., 2003) found that co-morbid AUDs in patients with MDD have memory impairment on the behavior. The lateral habenula (LHb) plays a very important role in memory, and interestingly enough, both AUDs (Mathis and Lecourtier, 2017) and MDD (Park et al., 2017) show dysfunction of the LHb. These studies suggest lesions of memory and related brain area LHb might be a reason of this comorbidity. The LHb is a core brain region related to memory process and adaptive behavior selection (Lee and Huang, 1988; Goutagny et al., 2013). First, a large number of animal studies have shown the LHb is in a key position to process memory as it is connected with the main networks mediating such a function. The LHb is indirectly connected with the dorsal hippocampus (dHPC) (Goutagny et al., 2013) and projects to the mediodorsal nucleus of the thalamus (Araki et al., 1988). Moreover, it is involved in modulating hormones associated with memory, such as dopamine (Brown and Shepard, 2016), serotonin (Kalén et al., 1990), as well as raphe serotonergic neurons (Wang and Aghajanian, 1977). Third, recently, different studies have suggested that the LHb may take part in memory processes and perhaps play an important role in the selection of the most adapted “memory-based behavior” (Mathis and Lecourtier 2017).

We know that the patients with depression and AUD have more stressful events that are more prone to negative emotions. Consequently, given the well-known detrimental effects of altered stress coping on learning and memory (Quervain et al., 2017), the marked memory impairments observed could partly be the consequence of such a failure to cope with stress. The LHb has been involved in not only participating in the online processing of contextual information but also the processing of the emotional valance of incoming information in order to adapt to particularly stressful situations. Then, they produce risky behaviors such as drinking.

In short, negative emotion, deficient cognitive control and dysfunction of the LHb may increase the risk of developing alcohol-seeking behavior, potentially as a result of overlapping neural mechanisms.

## Personality disorders and AUDs

PDs are highly comorbid with AUDs in both general and clinical populations (Trull et al., 2000), especially in cluster-B alcoholics (Mccarter et al., 2016). The strongest and most consistent links reported in the literature have been found between externalizing-related personality pathology (antisocial personality disorder [ASPD] and borderline personality disorder [BPD]) and AUDs (Jahng et al., 2011). Approximately 50–70% of psychiatric inpatients with BPD also meet diagnostic criteria for substance use disorders, most commonly alcohol use disorder (Zanarini et al., 2004), The study found the patients who met the DSM criteria for ASPD were 21 times more likely to develop alcohol abuse and dependence at some point during their lives than were the people who did not have ASPD (Regier et al., 1990).

Studies on cognitive function suggest that alcohol-seeking in PDs may be associated with impulsivity (Trull et al., 2000). Self-report measures of impulsivity were higher in cluster-B alcoholics than in alcoholics without PD (Dom et al., 2006). Impulsivity in the early years is related to greater risk of future alcohol dependence, and adults with high impulsivity scores are more likely to be diagnosed with alcoholism (Prince van Leeuwen et al., 2011). Impulsivity increases the vulnerability to AUDs through negative emotion (Shin et al., 2015), worse cognitive performance (Haaland et al., 2009), and impaired executive function (Stevens et al., 2003). The study also found that AUDs was strongly and negatively correlated with BPD, by contributing to unemployment, poor school performance, promiscuity (Gregory et al., 2008) and neuroticism–negative affectivity (Trull et al., 2004).

Impulsivity predicts development of AUDs, which is higher in family-history positive (FHPs) with more alcohol-dependent relatives (Dick et al., 2010). Neuroimaging Go/No-Go studies have identified functional abnormalities consistent with increases in aspects of impulsivity (e.g., poor response inhibition) in FHPs. But there is no consensus on the brain regions of alcohol—related impulses, and these studies have different conclusions. The study finds that bilateral junction of the anterior insula and inferior frontal gyrus (insula/IFG) were associated with impulsivity and addiction and Go/No-Go task activation (Stevens et al., 2007). The insula/IFG is integral to response inhibition (Stevens et al., 2007). The insula has been proposed to be relevant to addiction, given its role in interoception and integration of information from regions important for cognitive-control and affective processes (Naqvi and Bechara, 2010).

In a previous study that utilized SPECT, alcoholics with a comorbid diagnosis of ASPD exhibited reductions in regional cerebral blood flow within the frontal lobes (Vural, 1996). In another study (Soloff et al., 2003), PET technology was used to demonstrate reduced regional glucose metabolism in the prefrontal cortex (orbitofrontal, anterior medial, superior frontal). These studies show that the prefrontal lobe is the key part of comorbid the cluster-B PDs in patients with AUDs, which are indeed associated with addiction, especially inferior frontal gyrus (IFG).

Devito (Devito et al., 2013) thinks that activity in the left insula/IFG cluster correlated with aspects of impulsivity, which, in turn, were associated with alcohol use measures. His study also confirms this view when successfully inhibiting responses on “No-Go” trials, FHPs activated the left insula/IFG region more robustly than FHNs (Devito et al., 2013).

In summary, impulsivity is the obvious characteristic of Cluster B personality disorder, further impulsivity is associated with abnormality of the prefrontal lobe (especially in IFG), having an injury in IFG of Cluster B personality disorder might be risk factors for alcohol-seeking behavior.

## Conclusions

The close relationship between psychiatric disorders (e.g., Schizophrenia, mood disorders, PDs) and AUDs suggests that psychiatric disorders are predisposing factors for AUDs. In this review, we investigated three comorbid disorders with AUDs, focusing on cognitive function in these disorders and neural imaging studies. We found that memory deficits, cognitive control, negative emotion, impulsivity, and affective instability may increase an individual's vulnerability to AUDs. This review may indicate the neural basis and clinical subdivisions of AUDs, as well as suggest new approaches for the preclinical treatment of AUDs.

## Author contributions

PY and XZ work for substantial contributions to the conception or design; YW work for drafting the work or revising it critically for important intellectual content; RT and CH work for final approval of the version to be published; CH contribution for refining the ideas; SL contribution for looking up the literature for this article.

### Conflict of interest statement

The authors declare that the research was conducted in the absence of any commercial or financial relationships that could be construed as a potential conflict of interest.
